# Association between the APOB XbaI and EcoRI polymorphisms and lipids in Chinese: a meta-analysis

**DOI:** 10.1186/s12944-015-0125-z

**Published:** 2015-10-07

**Authors:** Wei Gu, Mingduo Zhang, Shaojun Wen

**Affiliations:** Department of Hypertension Research, Beijing Anzhen Hospital, Capital Medical University and Beijing Institute of Heart Lung and Blood Vessel Diseases, 2 Anzhen Road, Beijing, 100029 People’s Republic of China; Department of cardiology, Beijing Anzhen Hospital, Capital Medical University, Beijing, People’s Republic of China

**Keywords:** Apolipoprotein B or APOB, Chinese, Lipid, Polymorphism

## Abstract

**Background:**

No previous meta-analysis was to report the association between the apolipoprotein B (APOB) XbaI and EcoRI polymorphisms and serum lipids in Chinese. We performed the study to investigate their potentially association.

**Methods and Results:**

Studies in English and Chinese were found via a systematic search of Pubmed, Embase, CNKI and Wanfang databases. The dominant genetic model and random-effects model were used to pool data from individual studies. As a result, a total of 30 articles with 5611 subjects for XbaI and 2653 subjects for EcoRI were included in the current study. For the XbaI polymorphism, overall, subjects carrying X+ allele were significantly associated with higher TC,TG and LDL compared with X-X- genotype (Pvalue =0.0006, OR (95 %) = -0.55 (-0.86,-0.23); Pvalue = 0.0004, OR (95 %) = -0.30 (-0.47,-0.14); (Pvalue = 0.05, OR (95 %) = -0.23(-0.46,-0.00), respectively). Similar results were observed in the subgroups of Han, healthy individuals (HT), coronary heart disease (CHD), cerebral infarction (CI), and cholelithiasis. For HDL, positive association between X+ allele with Lower lipid value was found in CHD and CI subgroups. For EcoRI polymorphism, overall, the E- allele carriers were found to be obviously linked with elevated LDL and lower HDL compared with E + E+ genotype (Pvalue = 0.02,OR (95 %) = -0.27 (-0.49,-0.05); Pvalue = 0.01, OR (95 %) = 0.17 (0.03, 0.30), respectively). TC was significantly high in subjects carrying E- allele in the subgroup of hyperlipidemia. No evidence of publication bias was observed.

**Conclusions:**

The two genetic variants of APOB may be associated with serum lipids in Chinese.

## Introduction

Many epidemiological studies have reported that metabolic disorders in serum lipids are considerable risk factors for premature coronary artery disease and atherosclerosis [1]. Serum lipids are multifactorial that emphasize the contribution of genetic as well as environmental factors. Genetically determined variation has been shown to have a close relationship with the metabolism abnormality of lipids and the pathogenesis of atherosclerosis [2, 3]. Although no convincing gene has been found to be of the importance for lipids, the obvious candidate genes are still necessary for a better understanding of lipid metabolism.

Apolipoprotein B (APOB) is an important protein component in contribution to intermediate density lipoprotein, the formation of very low density lipoprotein, low density lipoprotein particle, and is related to the clearance of LDL in serum. Beside these, it mediates cellular uptake of cholesterol and is the ligand that binds to the LDL receptor [4]. The gene coding for APOB has been cloned and is located on the short arm of chromosome 2 (q23q24). Many variants of the APOB gene has been found to be directly linked with lipid levels. In our study, among these variants, we paid particular attention to the most investigated XbaI (rs693 ) in exon 26 and EcoRI (rs1042031) in exon 29 single nucleotide polymorphisms in the APOB gene.

A meta-analysis by Boekholdt et al. [5] ten years ago had showed the relationship between the XbaI and EcoRI polymorphisms and lipids in Caucasian subjects. However, no meta-analysis about their association has concentrated on Chinese up to now. The ethnic difference in genetic background between Caucasian and Chinese may result in different findings. Moreover, the results of many published papers for the two polymorphisms in Chinese were inconclusive. Some of them showed strong associations between the XbaI and EcoRI polymorphisms in the APOB gene and serum lipids, whereas others had null association. Therefore, we performed a systematic meta-analysis of all eligible studies investigating the APOB XbaI and EcoRI polymorphisms and their association with serum lipids in Chinese.

## Methods

### Literature search and selection criteria

To identify the studies evaluating the relationship between the APOB XbaI and EcoRI polymorphisms and lipid profile in Chinese, a computerized literature search of PubMed, EMBASE, Wanfang and CNKI databases was carried out, applying the following keywords: ‘apolipoprotein B or APOB’, ‘polymorphism’, ‘lipid’, ‘Chinese or China or Taiwanese or Taiwan’ (up to September 1, 2015). The articles in English and Chinese would be included. References of the relevant articles were also examined. If multiple articles used the same data, only the more detailed one was selected. The included studies should be accordance with the later criteria: (i) studies evaluating the association of the APOB XbaI and EcoRI polymorphisms with lipids in Chinese, (ii) At least one of the lipid phenotypes was measured: total cholesterol (TC), triglyceride (TG), low density lipoprotein(LDL), and high density lipoprotein(HDL), (iii) Sufficient data involving genotype frequency as well as mean and standard deviation (SD) of lipids, (iv) If the article was a retrospective case-control study, each population (case/control groups) was treated as a single study and genotype frequency among control populations must be tested for Hardy-Weinberg equilibrium (HWE). If the genotype frequency was not supplied or calculated, the writers of these papers would be contacted by emails.

### Data extraction

Two of the authors extracted the information from each study independently. Inconsistencies were discussed between the authors to reach an agreement. For each study, the following information would be collected: first author, year, ethnicity, resident region, detection method, sample size, character of participants, and lipid phenotypes of each study.

### Statistical analysis

In our study, standardized mean difference (SMD) with their 95 % CIs were used to measure the strength of the association of the APOB XbaI and EcoRI polymorphisms with lipids. For the two polymorphisms, we tested the dominant genetic model (X-X- vs. X + X-/X + X+, E + E+ vs. E + E-/E-E-), because the low frequency of homozygosity for the mutant alleles would yield a large number of studies with zero cell counts, leading to the unreliable estimates, and the X + X-/X + X+ and E + E-/E-E- genotypes in most studies were combined into one group. The random-effects model was used to assess the pooled estimates and the significance of the pooled estimates was determined using a Z-test. Heterogeneity among studies was assessed by the X^2^-based Q-statistic test and considered significant for *P* < 0.10 [6]. Subsequently based on racial ground, subgroup analysis was undertaken for Han and non-Han minority in Chinese. In addition, subgroup analysis for the XbaI polymorphism was also planned for healthy individuals (HT), coronary heart disease (CHD), cerebral infarction(CI), hyperlipidemia and cholelithiasis. Subgroup analysis for the EcoRI polymorphism was performed for healthy individuals (HT), coronary heart disease (CHD) and hyperlipidemia. Each subgroup had at least three independent studies. Sensitivity analysis was carried out by removing a single study each time in order to find out whether any single study could bias the overall estimate. Sensitivity analysis were used to detect the cause of heterogeneity when the between-study heterogeneity would exist. Finally, we evaluated publication bias by funnel diagram. The funnel diagram asymmetry, suggesting publication bias, was precisely assessed by Egger’s linear regression test and Begg’s rank correlation test [7, 8] we re-calculated HWE using a web program (http://ihg.gsf.de/cgi-bin/hw/hwal.pl). All data were analyzed with Review Manager 5.0 (Oxford, UK) and STATA 12.0 (Stata Corp., TX, USA). All *P* values reported were two-sided, and a significance level of less than 0.05 was considered statistically significant.

## Results

### Inclusion and characteristics of studies

As a beginning analysis, 77 relevant articles were found after literature search and selection. Among these selected studies, 43 articles were removed because their data were overlapping or duplicated. One paper [9] was deleted due to meta-analysis and two [10, 11] were excluded due to lack of the available information. For the EcoRI polymorphism, the data from 3 studies [12–14] was excluded as they deviated from HWE. Finally, the remaining 30 articles [13–42] with 5611 subjects for XbaI and 2653 subjects for EcoRI were included in this meta-analysis. Among them, Fan et al. [18] was an unpublished theses from the dissertation database, that is, an open sub-database shared by Wanfang and CNKI. 14 articles [14–16, 20, 22, 24, 27, 28, 30–34, 36] provided the available data on case and controls, which would be respectively handled as the separate two studies for each analysis. For all eligible studies, HWE had been recounted and had to be obeyed. The characteristics of the included studies were showed in Table 1.

**Table 1:** Main characteristics of the included studies in the meta-analysis

First author,year	Ethnicity	Region	Sample size	SNPs	Character of subjects	Method	Lipid profile
Bai, [15]2008	Han	Ningxia	165	EcoRI	CA/HT	PCR-RFLP	TC,TG,LDL,HDL
Chai, [16] 1996	Han	Shanghai	82	XbaI	CI/HT	PCR-RFLP	TC,TG,LDL,HDL
Evans, [17] 1993	Han	Shanxi	143	XbaI	HT	PCR-RFLP	TG,LDL,HDL
Fan, [18] 2009	Han	Fujian	387	XbaI	Hyperlipidemia	PCR-RFLP	TC,TG,LDL,HDL
Guo, [19] 1996	Han	Liaoning	55	XbaI	CI	PCR-RFLP	TC,TG,LDL,HDL
Han, [20] 2000	Han	Shanghai	631	XbaI	Cholelithiasis/HT	PCR-RFLP	TC,TG,LDL,HDL
Hu, [21] 2008	Han	Guangxi	150	XbaI	PNS	PCR-RFLP	TC,TG,LDL,HDL
Hu, [13] 2009	Han	Guangxi	200	XbaI	HT	PCR-RFLP	TC,TG,LDL,HDL
Ji,[22] 2014	Han	Inner mongolian	120	EcoRI,XbaI	Cholelithiasis/HT	PCR-RFLP	TC,TG,LDL,HDL
Li, [23] 1997	Han	Tianjing	71	EcoRI,XbaI	CHD	PCR-RFLP	TC,TG,LDL,HDL
Liu, [24] 2008	Han	Hunan	230	EcoRI	CI/HT	PCR-RFLP	TC,TG,LDL,HDL
Liu, [25] 2010	Han	Shanghai	186	XbaI	Gastric cancer	PCR-RFLP	TC,LDL
Liu,[26] 2014	Li/Han	Hainan	351	XbaI	HT	PCR-RFLP	TC,TG,LDL,HDL
Ma, [27] 2012	Yao	Guangdong	500	EcoRI,XbaI	Hyperlipidemia/HT	PCR-RFLP	TC,TG,LDL,HDL
Pan, [28] 1995	Han	Taiwan	301	EcoRI,XbaI	CHD/HT	PCR-RFLP	TC,TG,LDL,HDL
Saha, [29] 1992	Han	Singapore	196	EcoRI,XbaI	HT	PCR-RFLP	TC,TG,LDL,HDL
Tan, [30] 2003	Han	Jiangsu	211	EcoRI,XbaI	Cholelithiasis/HT	PCR-RFLP	TC,TG,LDL,HDL
Wang, [31] 1999	Han	Beijing	377	XbaI	CI/HT	PCR-RFLP	TC,TG,LDL,HDL
Wei, [32] 2001	Han	Sichuan	169	XbaI	Cholelithiasis/HT	PCR-RFLP	TC,TG,LDL,HDL
Xie, [33] 2010	Han	Xinjiang	300	EcoRI,XbaI	Hyperlipidemia/HT	PCR-RFLP	TC,TG,LDL,HDL
Yan, [34] 2003	Han	Beijing	257	EcoRI	CHD/HT	PCR-RFLP	TC,TG,LDL,HDL
Yao, [14] 1999	Han	Jiangsu	141	XbaI	CI/HT	PCR-RFLP	TC,TG,LDL,HDL
Yao, [35] 2005	Han	Xinjiang	112	EcoRI,XbaI	fatty liver	DNA chips	TC,TG,LDL,HDL
Ye, [36] 1995	Han	Beijing	203	XbaI	CHD/HT	PCR-RFLP	TC,TG,LDL,HDL
Ye, [37] 2003	Han	Beijing	88	XbaI	Hyperlipidemia	PCR-RFLP	TC,TG,LDL
Zhang, [39] 2009	Han	Hunan	130	XbaI	CI	PCR-RFLP	TC,TG,LDL,HDL
Zhang,[38] 2010	Han	Xinjiang	154	EcoRI	CHD	PCR-RFLP	TC,TG,LDL,HDL
Zhang, [40] 2015	Han	Beijing	82	EcoRI,XbaI	Hyperlipidemia	PCR-RFLP	TC,TG,LDL,HDL
Zhao, [41] 1997	Han	Beijing	117	XbaI	CI	PCR-RFLP	TC,TG,LDL,HDL
Zhu, [42] 2001	Han	Beijing	308	XbaI	HT	PCR-RFLP	TC,TG,LDL,HDL

*CA* carotid atherosclerosis, *HT* healthy individuals, *CI* cerebral infarction, *PNS* primary nephrotic syndrome, *CHD* coronary heart disease, *PCR-RFLP* polymerase chain reaction-restriction fragment length polymorphism, *TC* total cholesterol, *TG* triglyceride, *LDL* low density lipoprotein, *HDL* high density lipoprotein

In our study, based on the available genotype and allele frequency, we observed that, the X+ allele frequency of XbaI was 7.0 % in the whole population, and the E- allele frequency of EcoRI was 8.0 %, which both obviously had a lower frequency of the mutant allele than the Caucasian population.

### Association of the XbaI polymorphism with various lipids

The results of the relationship between the APOB XbaI ploymorphism and lipids in Chinese were shown in Table 2. In this analysis of TC, 5468 subjects (37 studies) were included. Overall, we observed that, TC was significantly high in subjects carrying X+ allele compared with X-X- genotype (*P*_value_ = 0.0006, OR (95 %) = −0.55 (−0.86,−0.23)). Similar findings were seen in the subgroups of Han (*P*_value_ = 0.001, OR (95 %) = −0.59 (−0.94,−0.24)), CI (*P*_value_ = 0.007, OR (95 %) = −0.85 (−1.46,−0.24)) and cholelithiasis (*P*_value_ = 0.004, OR (95 %) = −0.54 (−0.91,−0.18)). However, no positive association was obtained in the subgroups of non-Han minorities, HT, CHD, and hyperlipidemia. For the TG analysis, 5425 subjects (37 studies) were collected. In the whole population, X+ allele carriers were found to be obviously associated with higher TG value compared with X-X- genotype (*P*_value_ =0.0004, OR (95 %) = −0.30 (−0.47,−0.14)). Similar results were observed in the subgroups of Han (*P*_value_ = 0.0009, OR (95 %) = -0.32 (-0.50,-0.13)), HT (*P*_value_ = 0.05, OR (95 %) = −0.31 (−0.62,0.00)) and CHD (*P*_value_ = 0.009, OR (95 %) = −0.53 (−0.93,−0.13)). There was no evidence of correlation in the subgroup of non-Han minorities, CI, cholelithiasis and hyperlipidemia.

**Table 2:** Overall and subgroup associations of the APOB XbaI polymorphism and lipids

Lipids	Overall or subgroups	Studies	Subjects	OR (95 %)	*P*_value_	P_heterogenity_	P_e_
TC	Overall	37	5468	−0.55[−0.86,−0.23]	0.0006	<0.01	0.08
	Han	34	4812	−0.59[−0.94,−0.24]	0.001	<0.01	
	Non-Han minorities	3	651	−0.25[−0.61, 0.10]	0.16	0.08	
	HT	16	2708	−0.61[−1.3, 0.07]	0.08	<0.01	
	CHD	3	322	0.02[−0.37, 0.42]	0.90	0.71	
	CI	6	577	−0.85[−1.46,−0.24]	0.007	<0.01	
	Hyperlipidemia	5	957	−0.24[−0.71, 0.22]	0.31	<0.01	
	Cholelithiasis	4	456	−0.54[−0.91,−0.18]	0.004	0.11	
TG	Overall	37	5425	−0.30[−0.47,−0.14]	0.0004	<0.01	0.25
	Han	34	4774	−0.32[−0.50,−0.13]	0.0009	<0.01	
	Non-Han minorities	3	651	−0.19[−0.41, 0.02]	0.08	0.98	
	HT	17	2851	−0.31[−0.62, 0.00]	0.05	<0.01	
	CHD	3	322	−0.53[−0.93,−0.13]	0.009	0.41	
	CI	6	577	−0.50[−1.02, 0.02]	0.06	0.0001	
	Hyperlipidemia	5	957	−0.29[−0.62, 0.04]	0.09	0.01	
	Cholelithiasis	4	456	−0.11[−0.34, 0.12]	0.36	0.86	
LDL	Overall	38	5611	−0.23[−0.46,−0.00]	0.05	<0.01	0.80
	Han	35	4960	−0.23[−0.49, 0.03]	0.09	<0.01	
	Non-Han minorities	3	651	−0.26[−0.47,−0.04]	0.02	0.5	
	HT	17	2851	−0.21[−0.60, 0.18]	0.29	<0.01	
	CHD	3	322	0.15[−0.25, 0.54]	0.47	0.6	
	CI	6	577	−0.52[−1.08, 0.05]	0.07	<0.01	
	Hyperlipidemia	5	957	0.15[−0.47, 0.78]	0.63	<0.01	
	Cholelithiasis	4	456	−0.4[−0.64,−0.17]	0.0007	0.46	
HDL	Overall	36	5337	0.17[−0.08, 0.41]	0.18	<0.01	0.86
	Han	33	4686	0.15[−0.12, 0.43]	0.27	<0.01	
	Non-Han minorities	3	651	0.24[0.01, 0.47]	0.04	0.33	
	HT	17	2851	0.11[−0.43, 0.64]	0.07	<0.01	
	CHD	3	322	0.50[0.11, 0.90]	0.01	0.78	
	CI	6	577	0.43[0.13, 0.74]	0.005	0.11	
	Hyperlipidemia	4	869	0.10[−0.08, 0.27]	0.27	0.97	
	Cholelithiasis	4	456	−0.10[−0.33, 0.13]	0.40	0.78	

*TC* total cholesterol, *TG* triglyceride, *LDL* low density lipoprotein, *HDL* high density lipoprotein, *HT* healthy individuals, *CHD* coronary heart disease, *CI* cerebral infarction

*P*_value_: the significance of the pooled estimate (95 % confidence interval)

P_heterogeneity_: the Q statistic for heterogeneity

P_e_: Egger’s statistic for publication bias

All results were calculated under the dominant genetic model (X-X- vs. X + X-/X + X+)

**Fig. 1:**
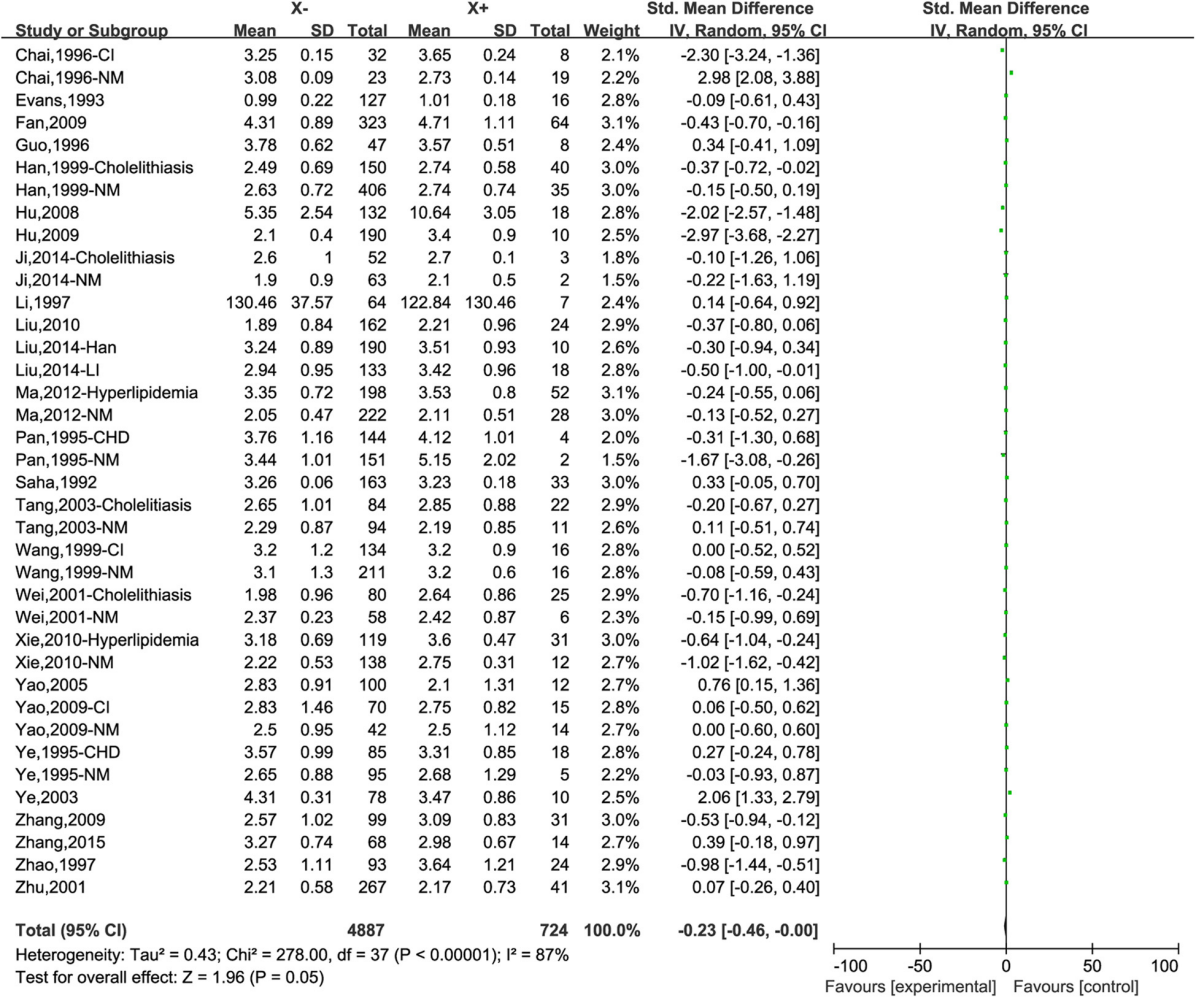
Association between the APOB XbaI polymorphism and LDL under the dominant genetic model (X-X- vs. X + X-/X + X+). “X-“ represents the X-X- genotype; X+ represents the X + X-/X + X+ genotypes

For LDL, 5611 subjects (38 studies) were collected. Overall, a marginally positive association between X+ allele with higher lipid value was found (*P*_value_ = 0.05, OR (95 %) = −0.23 (−0.46,−0.00), Fig. 1). In the subgroup analysis, we also observed the significant association in non-Han minorities (*P*_value_ = 0.02, OR (95 %) = −0.26 (−0.47,−0.04)) and cholelithiasis (*P*_value_ = 0.0007, OR (95 %) =−0.4 (−0.64,-0.17)). No statistically significant differences were found in the subgroups of Han, HT, CHD, CI and hyperlipidemia. Finally for HDL, 5337 subjects (36 studies) were analyzed. No significant association was observed in the overall and in the subgroups of Han, HT, hyperlipidemia and cholelithiasis. In the non-Han minorities, CHD and CI subgroups, subjects carrying X+ allele were associated with lower HDL value than X-X- (*P*_value_ = 0.04, OR (95 %) = 0.24 (0.01, 0.47); *P*_value_ = 0.01, OR (95 %) = 0.50 (0.11, 0.90; *P*_value_ = 0.005, OR (95 %) = 0.43 (0.13, 0.74), respectively ).

### Association of the EcoRI polymorphism with various lipids

**Fig. 2:**
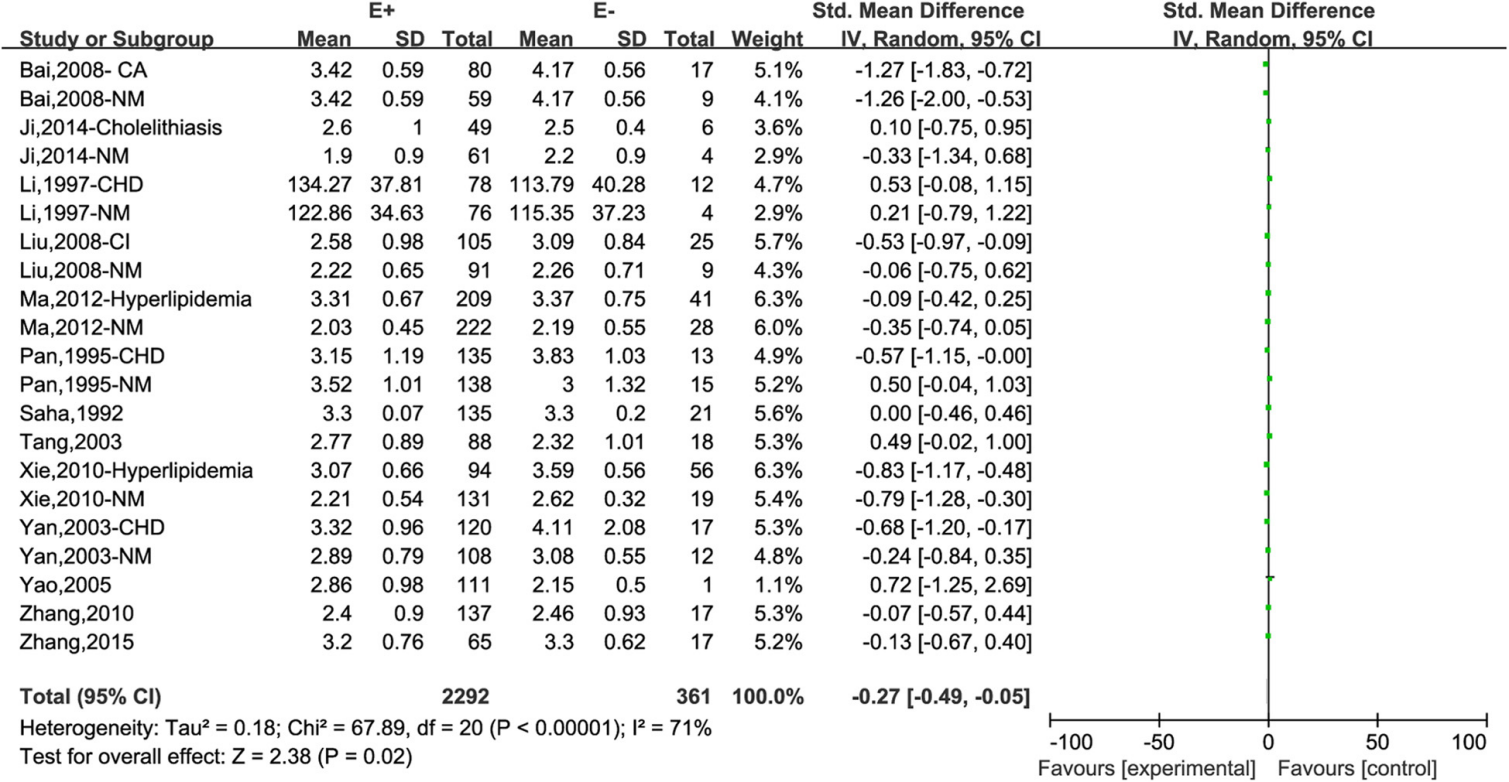
Association between the APOB EcoRI polymorphism and LDL under the dominant genetic model (E + E+ vs. E + E-/E-E-). “E + “ represents the E + E+ genotype; E- represents the E + E-/E-E- genotypes

The results of the relationship between the APOB EcoRI ploymorphism and lipids in Chinese were shown in Table 3. For TC, 2653 subjects (21 studies) were included. A significant association of the E- allele with higher TC was detected in the subgroup of hyperlipidemia (*P*_value_ < 0.01, OR (95 %) = −0.56 (−0.78,−0.35)). There was no difference in the whole population and in the subgroups of Han, HT and CHD. For TG, 2653 subjects (21studies) were assessed. We found no significant association between the EcoRI ploymorphism and TG in overall and even in the subgroup analysis. For LDL, 2653 subjects (21 studies) were found. Overall, subjects carrying the E- allele were found to be obviously linked with higher LDL compared with E + E+ genotype (*P*_value_ = 0.02, OR (95 %) = −0.27 (−0.49,−0.05), Fig. 2). Similar result was also shown in the subgroup of Han (*P*_value_ = 0.04, OR (95 %) = −0.28 (−0.53,−0.02)). In the subgroups of HT, CHD and hyperlipidemia, no significant association was found. For the HDL analysis, 2653 subjects (21studies) were included. Overall, HDL was significantly low in subjects carrying the E- allele compared with E + E+ genotype (*P*_value_ = 0.01, OR (95 %) = 0.17 (0.03, 0.30)). However, in all the subgroups, there was no significant association.

**Table 3:** Overall and subgroup associations of the APOB EcoRI polymorphism and lipids

Lipids	Overall or subgroups	Studies	Subjects	OR (95 %)	*P*_value_	P_heterogenity_	P_e_
TC	Overall	21	2653	−0.28[−0.58,0.01]	0.06	<0.01	0.64
	Han	19	2153	−0.27[−0.61,0.07]	0.11	<0.01	
	HT	9	1142	−0.19[−0.89,0.52]	0.61	<0.01	
	CHD	4	529	−0.25[−0.56,0.05]	0.1	0.29	
	Hyperlipidemia	3	482	−0.56[−0.78,−0.35]	<0.01	0.45	
TG	Overall	21	2653	−0.14[−0.32,0.03]	0.11	0.003	0.62
	Han	19	2153	−0.14[−0.34,0.07]	0.19	0.001	
	HT	9	1142	−0.01[−0.35,0.34]	0.97	0.002	
	CHD	4	529	−0.26[−0.55,0.04]	0.09	0.33	
	Hyperlipidemia	3	482	−0.15[−0.49,0.18]	0.37	0.11	
LDL	Overall	21	2653	−0.27[−0.49,−0.05]	0.02	<0.01	0.51
	Han	19	2153	−0.28[−0.53,−0.02]	0.04	<0.01	
	HT	9	1142	−0.26[−0.59,0.07]	0.13	0.005	
	CHD	4	529	−0.21[−0.73,0.31]	0.43	0.01	
	Hyperlipidemia	3	482	−0.36[−0.88,0.15]	0.17	0.006	
HDL	Overall	21	2653	0.17[0.03, 0.30]	0.01	0.2	0.44
	Han	19	2153	0.13[−0.02, 0.28]	0.09	0.2	
	HT	9	1142	0.13[−0.09, 0.35]	0.25	0.25	
	CHD	4	529	0.24[−0.03, 0.52]	0.08	0.5	
	Hyperlipidemia	3	482	−0.03[−0.44,0.38]	0.89	0.04	

*TC* total cholesterol, *TG* triglyceride, *LDL* low density lipoprotein, *HDL* high density lipoprotein, *HT* healthy individuals, *CHD* coronary heart disease

P_value_: the significance of the pooled estimate (95 % confidence interval)

P_heterogeneity_: the Q statistic for heterogeneity

P_e_: Egger’s statistic for publication bias

All results were calculated under the dominant genetic model (E + E+ vs. E + E-/E-E-)

### Sensitivity analysis

In the sensitivity analysis, we deleted one study at a time and found whether there was a individual study affecting the pooled SMD and the between-study heterogeneity. For the XbaI polymorphism, the removal of any single study did not obviously affect the heterogeneity and it still exist. However, the overall pooled SMD of LDL was apparently changed with the elimination in turn of eight studies (Chai- HT et al. [16], Guo et al. [19], Li et al. [23], Saha et al. [29], Yao et al. [35], Ye-CHD et al. [36], Ye et al. [37], and Zhang et al. [40]) (data not shown). For the EcoRI polymorphism, no individual study had an obvious influence on the between-study heterogeneity. After removing Saha et al. [29] for TC as well as Pan-HT et al. [28] and Saha et al. [29] for TG, the related overall pooled SMDs were changed (data not shown).

Finally, no significant publication bias was revealed under the dominant genetic model for XbaI (*P* = 0.08 for TC, 0.25 for TG, 0.8 for LDL, 0.86 for HDL, Table 2) and for EcoRI (*P* = 0.64 for TC, 0.62 for TG, 0.51 for LDL, 0.44 for HDL, Table 3).

## Discussion

Some discrepancies could be caused by differences in ethnic background. Thus, we restricted the analysis to the Chinese population and performed a meta-analysis containing 30 articles with 5611 participants for XbaI and 2653 participants for EcoRI to more precisely understand the relationship between these polymorphisms and lipids in Chinese. So far this meta-analysis was the largest one investigating the association of these two polymorphisms in the AOPB gene with lipid level in Chinese. In the present study, we found that, X+ allele of the XbaI polymorphism was significantly associated with higher TC,TG and LDL in all subject, and E- allele of the EcoRI polymorphism was obviously associated with higher LDL and lower HDL level. Our results involving XbaI were partially consistent with the findings of the previous meta [5]. The difference on the results about EcoRI may be due to the distinct genetic backgrounds of the included populations.

The XbaI polymorphism within the coding region of APOB mRNA is caused by a silent cytosine to thymine mutation in the third base of the threonine codon at residue 2488 in the mature APOB protein, and this site itself is of dubious functional significance. Nevertheless, this polymorphism may be a genetic indicator in linkage disequilibrium with other functional mutations in the APOB gene or a nearby-located gene [43, 44], which can affect the metabolism of serum lipids. Compared with X-allele, X+ allele carriers with higher LDL may be explained by differing clearance by the receptor-mediated pathway of LDL catabolism, and with higher TC/TG may be attributable to the production of a relatively receptor-inactive APOB in the mutational group [45]. As a matter of fact, many studies have reported that, compared with the wild-type genotype, subjects with X+ allele have apparently higher TC,TG, APOB and LDL levels [5, 46, 47]. The APOB EcoRI polymorphism in the coding sequence could result in an acidic basic amino acid substitution, which is non-conservative and has putative importance in APOB protein function [48]. E+ allele carriers in association with abnormal lipid parameters were observed in many studies compared with E- allele carriers [5, 13]. Taken together, APOB plays a critical role in the lipid transport. If the gene encoding APOB has mutational changes, APOB structure and function would be affected and finally lead to lipid metabolism disorder, such as increase of plasma ApoB and LDL, decrease of HDL, etc.

For the Han subgroup, owing to the largest ethnic in Chinese and most related studies included in our meta, the association between the XbaI and EcoRI polymorphism and lipids was largely consistent with the results in the whole population. Irregular results involving the non-Han minorities in Chinese was seemingly understandable in a complex genetic background and limited number of studies. Studies in a larger population based on a single minority are needed for a more clear observation. For the subgroups of HT, CHD, CI and cholelithiasis in the XbaI analysis, our finding were partially in accordance with the published four articles [5, 9, 49-50]. In addition, hyperlipidemia was diagnosed when one of the following four lipids (TC、LDL、HDL and TG ) was more than the normal serum level, and thus the statistical power of the single lipid (such as LDL) to detect differences may be limited due to the smaller involved sample size. For EcoRI, when considering the subgroup analysis of hyperlipidemia, the conclusion may be influenced and should be treated with caution, because sample size of the subgroup as well as study number were relatively small (482 and 3, respectively).

Future related studies in this area should consider several limitations in the present meta-analysis. Firstly, a significant heterogeneity was detected in the analysis, and its contributors may be population source, study design, etc. Secondly, due to the limited number of studies at this time, the subgroup analysis on non-Han minority populations (such as Yao) can not be well done. Thirdly, the analysis did not focus on the detailed functional research of the studied two polymorphisms. Finally, beside genetic influence, the relationship between APOB single nucleotide polymorphisms and lipid profile was also affected by many environmental factors that didn’t be fully conducted in this study, which possibly confounded the results.

In conclusion, our meta-analysis showed significant association between the APOB XbaI polymorphism and lipids (TC, TG and LDL) in Chinese. Similar conclusions were observed in the subgroups of Han, HT, CHD, CI and cholelithiasis. For the AOPB EcoRI polymorphism, the E- allele carriers may be a genetic factor for higher LDL and lower HDL levels in Chinese. Additional studies with larger sample size should be conducted in Chinese to make clear the association of APOB SNPs with lipids. Studies investigating their detailed function were equally needed.

## Abbreviations

APOB: Apolipoprotein B
TC: Total cholesterol
TG: Triglycerides
LDL: Low-density lipoprotein
HDL: High-density lipoprotein
SD: Standard deviation
HWE: Hardy-Weinberg equilibrium
SMD: Standardized mean difference
HT: Healthy individuals
CHD: Coronary heart disease
CI: Cerebral infarction
SNP: Single-nucleotide polymorphism
CA: Carotid atherosclerosis
PNS: Primary nephrotic syndrome
